# A Challenging Uncommon Presentation of Steroid-Resistant Tumefactive Multiple Sclerosis: A Brainstem Crisis Resolved With Plasma Exchange and Ocrelizumab Maintenance

**DOI:** 10.7759/cureus.97852

**Published:** 2025-11-26

**Authors:** Kanchan Kanchan, Pawan Kumar, Vikram Kumar, Muskan Maheshwari

**Affiliations:** 1 Acute Medicine, University Hospitals Birmingham National Health Service (NHS) Foundation Trust, Birmingham, GBR; 2 General Internal Medicine, University Hospitals Birmingham National Health Service (NHS) Foundation Trust, Birmingham, GBR; 3 Emergency Medicine, Chelsea and Westminster Hospital National Health Service (NHS) Foundation Trust, London, GBR; 4 Internal Medicine, Heartlands Hospital, Birmingham, GBR

**Keywords:** brainstem lesions, demyelinating neurological disorder, s: multiple sclerosis, steroid-resistant multiple sclerosis, stroke mimic, tumefactive demyelination

## Abstract

Tumefactive multiple sclerosis (TMS) is a rare variant of multiple sclerosis that presents large demyelinating lesions, often mimicking neoplasms or stroke. We report the case of a 32-year-old man who presented with progressive right-sided weakness, facial involvement and cranial nerve deficits. Neuroimaging revealed a demyelinating lesion in the left pons with additional older plaques. Despite initial treatment with high-dose corticosteroids, the patient showed no improvement. Subsequent plasma exchange led to significant neurological recovery and ocrelizumab was initiated for relapse prevention. This case highlights the diagnostic challenge of TMS presenting as a brainstem syndrome and the effective role of plasma exchange followed by ocrelizumab maintenance therapy.

## Introduction

Tumefactive multiple sclerosis is an uncommon demyelinating disorder [1-4], accounting for less than 2% of all multiple sclerosis cases. It is characterized by large lesions greater than 2 cm [3,5], often associated with mass effect and contrast enhancement, which can mimic neoplastic, infectious or vascular pathologies. Diagnosis relies on MRI characteristics [5] and exclusion of other causes. Management typically begins with corticosteroids [2,6-8], though plasma exchange (PLEX) and immunotherapy may be considered [6-8] for refractory cases. Ocrelizumab, a humanized anti-CD20 monoclonal antibody [3,7,8,9], has shown efficacy in relapsing and primary progressive MS, but its role in TMS remains limited to a few cases.

## Case presentation

A 32-year-old gentleman presented with an eight-day history of gradually progressive right-sided sensory disturbance and weakness. Symptoms began as tingling and numbness in the right hand and foot, progressing to involve the entire right side of the body, including the face. He developed stiffness, difficulty walking and weakness affecting facial movements. He also reported tinnitus in the right ear, altered taste and mild swallowing changes, though no choking or visual disturbances. 

His examination revealed mild dysarthria, head was tilted to the right with a fixed right gaze and semi-flexed posture of the right limbs. Cranial nerve examination revealed reduced facial sensation, facial weakness, tongue deviation to the right, loss of taste on the left side and reduced hearing on the right. Up-gaze and down-gaze were preserved, though there was an inability of either eye to look left. No nystagmus or diploma was noted. Motor examination showed right-sided weakness (upper limb 0-2/5, lower limb 2/5 proximally, 0/5 distally), increased tone and brisk reflexes. The left side was normal. Sensation was markedly reduced to fine touch, pain and temperature on the right side, absent in the right lower limb and normal on the left.

**Figure 1:**
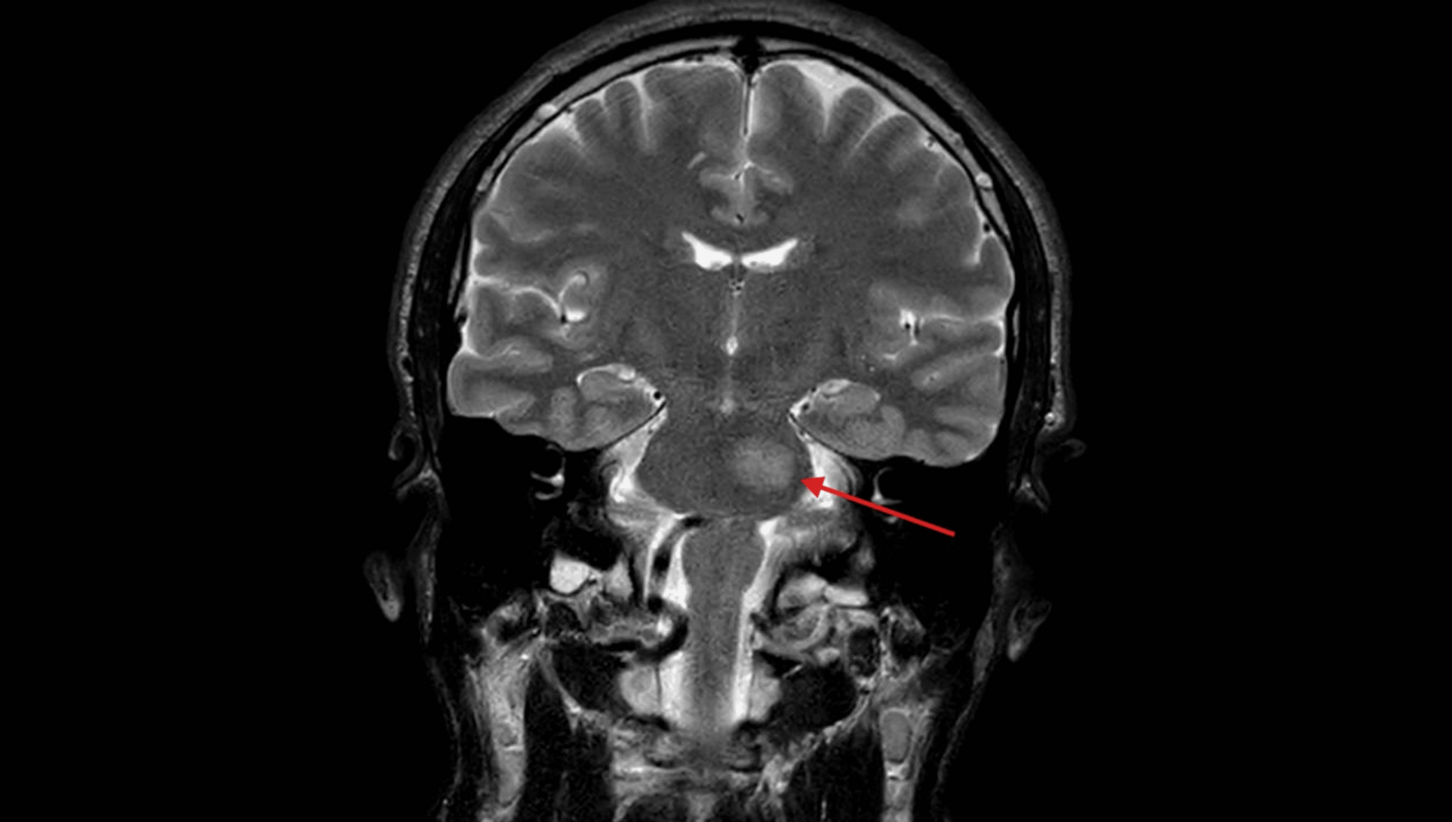
Coronal T2-weighted MRI showing a T2-hyperintense, expansile demyelinating lesion in the right pons, as indicated by the red arrow. The lesion extends toward the right middle cerebellar peduncle.

**Figure 2:**
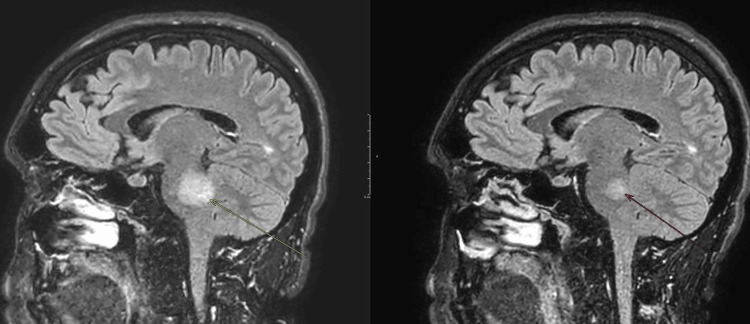
MRI of the brain shows significant expansion of the left pontine lesion, which demonstrates diffusion restriction at its margins. These appearances overall remain in keeping with acute active demyelination.

Initial MRI brain revealed a demyelinating lesion in the left pons with additional older white matter plaques. (Figure 1). Follow-up MRI after intravenous methylprednisolone demonstrated lesion expansion with diffusion restriction but no contrast enhancement, consistent with active demyelination (Figure 2). Cerebrospinal fluid (CSF) analysis was clear, with no evidence of infection or oligoclonal bands. Antibody testing for N-methyl-D-aspartate (NMDA) receptor, myelin oligodendrocyte glycoprotein (MOG) and aquaporin-4 was negative. Extensive metabolic screening, including homocysteine, methylmalonic acid and vitamins B1, B6 and biotin, all were unremarkable.

He received five days of high-dose intravenous methylprednisolone without neurological improvement. Two days after completing steroid therapy, a five-day course of plasma exchange was initiated. Clinical improvement was first noted on the third day of PLEX, with enhanced speech clarity, increased engagement and improved motor effort. Subsequently, Ocrelizumab was initiated as maintenance therapy to prevent relapses. He was referred for ongoing neurorehabilitation, including physiotherapy, occupational therapy and speech and language therapy, with continued functional improvement.

## Discussion

Tumefactive multiple sclerosis remains a diagnostic and therapeutic challenge due to its rarity and variable presentation. Brainstem involvement, as seen in this case [1,2,3,8], can mimic acute ischemic stroke or neoplastic processes. MRI findings of large demyelinating lesions [3,5,10] with incomplete ring enhancement and lack of mass effect are helpful distinguishing features (Figure 1). High-dose corticosteroids are first-line therapy [2,7,8]. However, in steroid-refractory cases, plasma exchange has shown significant benefit [6-8]. Ocrelizumab, though primarily indicated [3,6,8,9] for relapsing and primary progressive MS, is increasingly being reported as an effective maintenance therapy for TMS, potentially reducing relapse rates and lesion recurrence.

In this case, the patient’s initial steroid resistance, subsequent improvement with plasma exchange and stability maintained on ocrelizumab initiation supports a stepwise escalation approach [7,8,11] in the management of severe demyelinating events. This aligns with recent case series suggesting early initiation of immunotherapy [3,8,11] after acute recovery may improve long-term outcomes.

## Conclusions

This case highlights the importance of considering demyelinating pathology in patients presenting with progressive brainstem symptoms. Timely neuroimaging and multidisciplinary management are crucial. Plasma exchange remains a valuable treatment for steroid-unresponsive demyelination, while ocrelizumab may serve as an effective long-term maintenance option in tumefactive MS.

Beyond its diagnostic and therapeutic relevance, this case underscores the importance of individualized management strategies in severe demyelinating syndromes. Early recognition and a structured escalation approach from corticosteroids to plasma exchange and finally to targeted B-cell therapy can significantly improve outcomes and functional recovery. Continued reporting of such cases will contribute to refining therapeutic algorithms and enhancing evidence-based management of tumefactive multiple sclerosis.

## References

[1] Lucchinetti CF, Gavrilova RH, Metz I (2008). Clinical and radiographic spectrum of pathologically confirmed tumefactive multiple sclerosis. Brain.

[2] Hardy TA, Chataway J (2013). Tumefactive demyelination: an approach to diagnosis and management. J Neurol Neurosurg Psychiatry.

[3] Suh CH, Kim HS, Jung SC, Choi CG, Kim SJ (2018). MRI findings in tumefactive demyelinating lesions: a systematic review and meta-analysis. AJNR Am J Neuroradiol.

[4] Vaišvilas M, Vilionskis A, Sasnauskaitė I, Petrosian D, Mickevičiūtė E, Giedraitienė N (2023). Tumefactive demyelinating disorders as stroke mimics: Description of cases and systematic review of the literature. Mult Scler Relat Disord.

[5] Sánchez P, Chan F, Hardy TA (2021). Tumefactive demyelination: updated perspectives on diagnosis and management. Expert Rev Neurother.

[6] Fereidan-Esfahani M, Decker PA, Weigand SD (2023). Defining the natural history of tumefactive demyelination: a retrospective cohort of 257 patients. Ann Clin Transl Neurol.

[7] Al Malik YM (2024). Tumefactive demyelinating lesions: a literature review of recent findings. Neurosciences (Riyadh).

[8] Galetta K, Ham AS, Vishnevetsky A, Bhattacharyya S, Mateen FJ (2024). Disease modifying therapy in the treatment of tumefactive multiple sclerosis: A retrospective cohort study. J Neuroimmunol.

[9] Pervin I, Ramanathan S, Cappelen-Smith C, Vucic S, Reddel SW, Hardy TA (2024). Clinical and radiological characteristics and outcomes of patients with recurrent or relapsing tumefactive demyelination. Mult Scler Relat Disord.

[10] Qi F, Zhang Y, Li X, Fan J, Tan H, Quan C (2024). Tumor or demyelination? Three tumefactive multiple sclerosis case reports and literature review. World Neurosurg.

[11] Nagappa M, Taly AB, Sinha S (2013). Tumefactive demyelination: clinical, imaging and follow-up observations in thirty-nine patients. Acta Neurol Scand.

